# Pharmacological Inhibition of LSD1 for Cancer Treatment

**DOI:** 10.3390/molecules23123194

**Published:** 2018-12-04

**Authors:** Guan-Jun Yang, Pui-Man Lei, Suk-Yu Wong, Dik-Lung Ma, Chung-Hang Leung

**Affiliations:** 1 State Key Laboratory of Quality Research in Chinese Medicine, Institute of Chinese Medical Sciences, University of Macau, Macao 999078, China; yb67509@connect.um.edu.mo (G.-J.Y.); mb85817@connect.um.edu.mo (P.-M.L.); 2 Department of Chemistry, Hong Kong Baptist University, Kowloon Tong, Hong Kong 999077, China; sallywong@hkbu.edu.hk

**Keywords:** LSD1, demethylase, histone, breast cancer, prostate cancer, acute myeloid leukemia, cancer therapy

## Abstract

Lysine-specific demethylase 1A (LSD1, also named KDM1A) is a demethylase that can remove methyl groups from histones H3K4me1/2 and H3K9me1/2. It is aberrantly expressed in many cancers, where it impedes differentiation and contributes to cancer cell proliferation, cell metastasis and invasiveness, and is associated with inferior prognosis. Pharmacological inhibition of LSD1 has been reported to significantly attenuate tumor progression in vitro and in vivo in a range of solid tumors and acute myeloid leukemia. This review will present the structural aspects of LSD1, its role in carcinogenesis, a comparison of currently available approaches for screening LSD1 inhibitors, a classification of LSD1 inhibitors, and its potential as a drug target in cancer therapy.

## 1. Introduction

**Figure 1:**
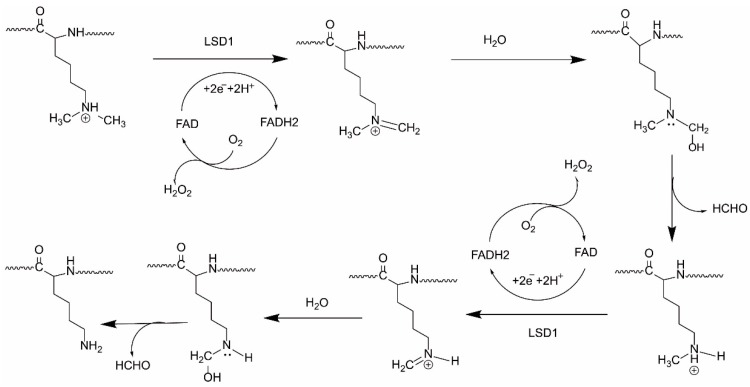
The catalytic mechanism of LSD1.

Lysine-specific demethylase 1A (LSD1), also named KDM1A and AOF2, is a flavin-dependent demethylase that was first identified in 2004 [1]. LSD1 can remove di- and mono-methyl groups from the fourth and nine positions on histone 3 protein (H3K4me2/1 and H3K9me2/1), which results in transcriptional repression or activation, respectively [2,3,4,5]. LSD1 has also been documented to remove mono- and di-methylated lysine residues from non-histone protein substrates, such as p53 [6], DNMT1 [2], E2F1 [7], HIF-1α [8],and STAT3 [9]. Recently, an isoform of LSD1 named LSD1+8a was identified, which is produced by alternative splicing of LSD1 and is involved in neuronal differentiation in neuron cells via demethylating H3K9me2/1 [10,11]. The catalytic mechanism of LSD1 and its isoform involves the oxidation of FAD and consumption of O_2_, yielding H_2_O_2_ and HCHO (Figure 1). Among the FAD-dependent demethylases, LSD1 shows differential expression in adult tissues [12]. However, LSD1 was found to be abnormally overexpressed in a range of solid tumors and in acute myeloid leukemia (AML), where it inhibits differentiation, and enhances proliferation, invasiveness, and cell motility, and also worsens prognosis [13,14]. Therefore, LSD1 inhibition is a potential anti-cancer therapeutic strategy.

## 2. Structure and Function of LSD1

### 2.1. Structure of LSD1

**Figure 2:**
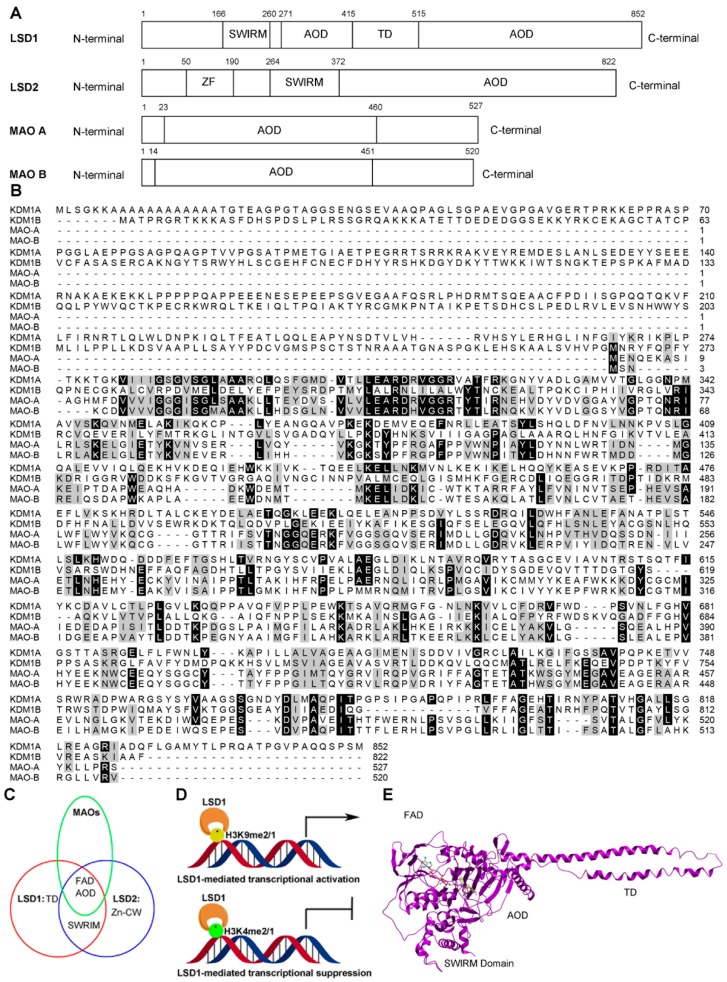
The structure and function of LSD1. (**A**) Domain architectures of four human FAD-dependent oxidation enzymes: LSD1, LSD2, MAO A, and MAO B. (**B**) Multiple alignment between amine oxidase domains of LSD family and MAOs from human beings. (**C**) Comparison of the domains of the LSD family and MAOs. FAD: FAD binding motif; TD: tower domain; Zn-CW: Zinc-finger domain. (**D**) LSD1-mediated transcriptional modulation. (**E**) Overall structure of LSD1 with trans-2-pentafluorophenylcyclopropylamine (2-PFPA); domains are labeled as in (**A**).

**Figure 3:**
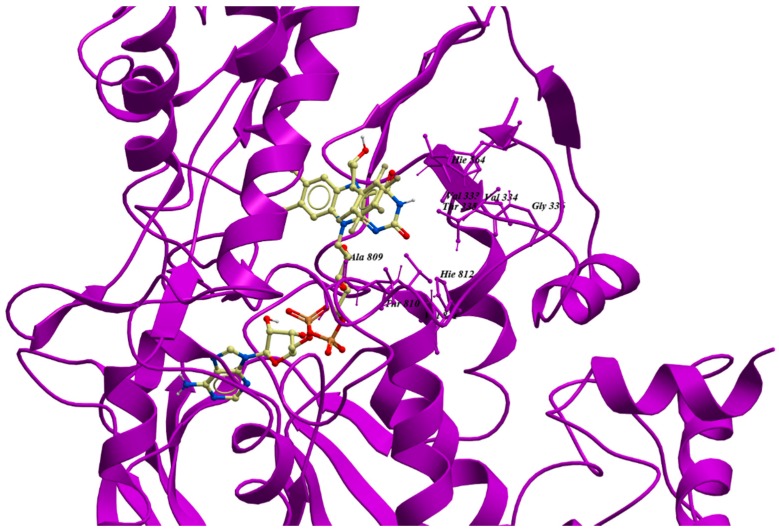
The structure of binding pocket of LSD1 with 2-PFPA. The image was generated from Molsoft ICM-pro 3.8–5 based on a previous report (PDB: 3ABT) [96].

To date, two families of lysine-specific demethylases (KDMs) have been reported: the Jumonji C family (JMJCs) and the LSD family. The JMJC family includes 19 members, and is dependent on α-ketoglutarate and Fe(II) to remove one to three methyl groups from lysine residues, while the LSD family comprises three members (LSD1, LSD1 + 8a, and LSD2) [15]. All the members of the LSD family are FAD-dependent oxidation enzymes, as their catalytic amine oxidase domain (AOD) requires FAD as a cofactor, similar to the monoamine oxidases A and B (MAOs) (Figure 2A,B). LSD1 and LSD2 contain a FAD-binding motif (FAD), the SWI3/Rac8/Moira (SWIRM) domain (LSD1: residues 166-260, LSD2: residues 264–372) and a catalytic AOD domain (LSD1: residues 272–415 and 516–852, LSD2: residues 373–822) (Figure 2A–C). However, differences between the two enzymes include that LSD2 possesses an N-terminal zinc finger domain (Zn-CW) (residues 50–190), while the AOD of LSD1 includes an insertion named the tower domain (residues 416–515) (Figure 2A,C) [1,16,17]. The *LSD1* gene encodes a construct of 852 amino acid residues, and the three key structural domains, the SWIRM domain, tower domain, and AOD domain, are highly conserved from yeast to human (Figure 2A,B) [18]. Unlike other SWIRM domains, the SWIRM domain of LSD1, which resembles a small alpha-helix, cannot bind to DNA, but is still able to take part in protein–protein interactions, which are imperative for regulating its functions of chromatin remodeling and histone modification [19,20]. The tower domain is a special domain protruding from AOD with two antiparallel helices, which is used by LSD1 to bind to RCOR1 and form the CoREST transcriptional repressor complex with other proteins [16,17].The AOD domain regulates enzymatic activity and targeting, and binds to several proteins [18]. The AOD comprises of two lobes, which are substantially more spacious and open than the corresponding regions of other FAD-dependent amine oxidases. The AOD of LSD1 consists of two well-defined subdomains: the FAD-binding motif (residues 272–356, 559–657, and 770–833) and the substrate recognition subdomain (residues 357–417, 523–558, and 658–769) (Figure 3) [21,22]. The FAD-binding motif is highly conserved among FAD-dependent oxidation enzymes, which may be responsible for the LSD1 inhibitory activity of some MAO inhibitors. The two subdomains form a big cavity, with the enzyme activity center at their interface [23]. In the tertiary structure of LSD1, the second lobe of the AOD is folded and adjacent to the SWIRM domain, where a hydrophobic binding pocket is formed between these two domains that allows LSD1 to encompass a large portion of the histone H3 tail (Figure 2E) [24]. This binding pocket also provides the structural basis for developing LSD1 inhibitors [25].

### 2.2. Functions of LSD1

LSD1 is involved in regulating many typical biological processes, including the epithelial-mesenchymal transition (EMT), stemness, cell motility, and through its ability to repress or activate transcription by demethylating H3K4me2/1 or H3K9me2/1, respectively (Figure 2C) [26]. LSD1 also demethylates some non-histone proteins and mediates the progression of some cancers [2,6,7,8,9].

#### 2.2.1. LSD1 as a Transcription Co-Repressor

Histone 3 methylation at the fourth site is a mark of transcriptional activation. LSD1 assembles together with several proteins into different complexes that demethylate H3K4me2/1 and shape chromatin into a repressive conformation. The repressive conformation may induce gene silencing via formation of the HP1/SU(VAR)3–9 or HOTAIR/PCR2 complexes, repress expression of specific genes in the form of core-BRAF35 or CoREST complexes [27,28], regulate stem cell properties via TLX and RCOR2 complexes [29,30,31], or perform nucleosome remodeling through NuRD complexes [17].

#### 2.2.2. LSD1 as a Transcription Co-Activator

LSD1 also functions as a transcriptional co-activator via facilitating the demethylation of H3K9me2/1. It can promote the transcription of hormone genes in prostate cancer cells and breast cancer cells through interacting with androgen and estrogen receptors [32,33]. As a co-activator, LSD1 also mediates the control of replication, imprinting and heterochromatin propagation.

#### 2.2.3. LSD1 as a Demethylase of Non-Histone Proteins

Apart from histone proteins, LSD1 can also remove mono- and di-methylation from non-histone proteins, which can be associated with cancer progression. For example, LSD1 increases the expression of glycolytic genes by increasing hypoxia-inducible factor-1a (HIF-1a)-mediated transcriptional activation [34]. LSD1 can also reduce the interaction of the tumor suppressor gene p53 and 53BP1 by removing a methyl group from p53K370me2, thus repressing p53 function [35]. Additionally, LSD1 regulates angiogenesis, cell cycle arrest, chromatin remolding and proliferation of cancer cells by demethylating HIF-1α [9], E2F1 [7], DNMT1 [3], and STAT3 [10]. A summary of LSD1 substrates and regulatory functions are presented in Table 1.

## 3. LSD1 and Cancer

### 3.1. LSD1 in Breast Cancer

Breast cancer is a cancer resulting from the malignant proliferation of mammary tissue [36,37]. Upregulation of LSD1 levels promotes ductal carcinomas in situ (DCIS) to evolve into invasive ductal carcinoma [38], and also accelerates development, proliferation, and metastasis of breast cancer cells [39]. When exposed to carcinogens, LSD1 will be upregulated and may promote occurrence of early stage breast cancer [40]. ERα, a major target for ER-positive breast cancer treatment, also relies on LSD1 demethylase activity to drive breast cancer [41]. ERα recruits LSD1 and other proteins to assemble a complex that binds to the promoters of estrogen-dependent and estrogen-responsive genes, thus promoting the proliferation of breast cancer cells [42,43,44,45]. However, CAC1 can interact with LSD1 and negatively regulate ERα function [46]. Conversely, ASXL2, another protein that can form a complex with LSD1, UTX, and MLL, mediates ERα activation to promote proliferation of breast cancer cells [47]. Moreover, LSD1 also cooperates with β-catenin to decrease the levels of the tumor suppressor protein Lefty1 in breast cancer [48]. LSD1 also controls breast cancer cell growth by interacting with histone deacetylases (HDACs) [49,50]. LSD1 can also maintain sensitivity to chemotherapy via coordinating with the SIN3A/HDAC complex in breast cancer [51]. Moreover, LSD1 has been linked with breast cancer metastasis. The Snail/Slug family of zinc finger transcription factors regulates epithelial–mesenchymal transition (EMT), which is critical for enhancing the invasiveness and motility of metastatic cells [52,53,54]. Studies have shown that Snail recruits LSD1 to epithelial gene promoters to demethylate H3K4me2, resulting in the silencing of key genes leading to cancer cell metastasis [55,56,57]. Moreover, Slug also interacts with LSD1 to enhance tumor metastasis [58]. Interestingly, LSD1 can also play a tumor repressive role in some complexes [59]. In the LSD1/NuRD (MTA3) complex, it can be recruited by the homeotic protein SIX3 to inhibit carcinogenesis and metastasis in breast cancer.

### 3.2. LSD1 in Prostate Cancer

The prostate is a critical gland in the male reproductive system, and the malignant proliferation of its tissues can lead to prostate cancer [60]. Studies have shown that LSD1 is closely correlated with cell proliferation, angiogenesis, migration and invasion in prostate cancer [61,62,63,64], including castrate-resistant prostate cancer (CRPC), which is characterized by resistance to androgen-deprivation therapy (ADT) [65]. LSD1 is overexpressed in CRPC and modulates expression of androgen receptor (AR)-independent or -dependent survival genes in CRPC cells in a demethylase-dependent manner [20,66,67,68,69,70]. In addition, LSD1 activates the lethal prostate cancer gene network connected with the binding protein ZNF217 [71]. Additionally, p53 interacts with LSD1 and modulates cell cycle and apoptosis of CRPC cells through LSD1 mediated-demethylation [6,20]. LSD1 was also reported to promote metastasis and invasion via raising PXN and LRPAR6 in androgen-independent prostate cancer cells [63].

### 3.3. LSD1 in AML

Acute myeloid leukemia (AML) is an abnormality of hematopoiesis featured by rapid self-renewal and proliferation in leukemic stem cells (LSCs), and suppression of normal hematopoietic stem cells (HSCs) [72]. LSD1 is a significant modulator of hematopoiesis and leukemogenesis [73,74,75]. LSD1 is overexpressed in HSC and early myeloblasts where it maintains stem cell self-renewal and regulates cell differentiation [75,76], via regulation of transcription factors and chromatin-modifying enzymes [13,77]. Studies showed that LSD1 downregulates stem cell and progenitor cell genes via H3K4 demethylation in promoter and enhancer regions, leading to regulation of hematopoietic differentiation, especially in the erythrocyte line [55,78,79,80,81]. LSD1 is also involved in the regulation of the progression of AML via different mechanisms from normal hematopoiesis and leukemogenesis [13,14]. In a mouse model of MLL-AF9 AML, LSD1 acted as an essential modulator of LSC differentiation [13]. The sustained expression of the MLL-AF9 oncogenic program is necessary for the potential of MLL-AF9 LSCs. LSD1 knockdown or chemical inhibition would impair MLL-AF9 cell differentiation and induce apoptosis in vitro and in vivo in a demethylase-dependent manner. Acute promyelocytic leukemia (APL), a subtype of AML, has been successfully treated using all-trans-retinoic acid (ATRA) to differentiate leukemic blasts. However, this compound is not effective in non-APL AML [82]. LSD1 inhibition could reactive the ATRA differentiation pathway in AML via upregulating H3K4me2 and myeloid-differentiation-associated genes, which suggests the feasibility of combination therapy using LSD inhibitors and ATRA. In a NOD-SCID γ mouse model, this combined therapy exhibited potential anti-leukemic effect [14]. Additional experiments further indicated that LSD1 can also regulate AML independent of its demethylase activity [83]. For example, inhibition of LSD1 significantly upregulated myeloid transcription-related genes without any significant genome-wide changes in H3K4me2/1 and H3K9me2/1 in the human AML cell line THP-1. Further studies showed that LSD1 was imperative to maintaining the myeloid differentiation block via binding to transcription factors, such as GFI1. Interestingly, the tranylcypromine (TCP)-derived LSD1 inhibitors could effectively overcome this block and restore differentiation independent of its histone demethylase activity.

## 4. Screening Methods for LSD1 Inhibitors

Given the crucial function of LSD1 in oncogenesis, discovering and developing novel LSD1 inhibitors may be a viable anti-cancer therapeutic strategy. Many screening methods have been reported recently to quickly and efficiently screen LSD1 inhibitors. Based on screening mechanism, the methods can also be divided into target-based assays, substrate-based assays, byproduct-based assays, and protein–protein interaction (PPI)-based assays. For target-based assays, virtual screening can be applied to quickly screen large numbers of ligands for LSD1 binding in silico [84]. Surface plasmon resonance (SPR) [85], isothermal titration calorimetry (ITC) [86] and bio-layer interferometry (BLI) [87] can also be used to monitor the interaction between LSD1 and its ligands. However, they are not suitable for high-throughput screening due to their requirement for expensive instrumentation, dedicated operation professionals, and complicated sample preparation steps [88]. For substrate-based assays, mass spectrometry (MS) is used to detect the truncated peptide substrates of LSD1 [89,90]. As the byproducts of LSD1-mediated demethylation, the production of H_2_O_2_ and/or HCHO is also as an indicator for LSD1 demethylase activity. Many byproduct-based assays have been developed, including the luminol coupled assay [91], ample red coupled assay [92], 4-aminoantipyrine coupled assay [93], and formaldehyde dehydrogenase (FDH) coupled assay [94]. Finally, PPI-based assays include the scintillation proximity assay and FRET-based assays. A comparison of different screening methods for LSD is presented in Table 2.

### 4.1. Target-Based Assay

Virtual screening has received attention as a versatile and effective tool for early-stage drug discovery and lead optimization [95]. To perform structure-based virtual screening, an X-ray crystal structure of LSD1 with an inhibitor is required. As an example, *trans*-2-pentafluorophenylcyclopropylamine (2-PFPA) can occupy the hydrophobic binding pocket between the second lobe of the AO domain and the SWIRM domain (Figure 2E). To achieve selectivity, 2-PFPA binds to FAD in the catalytic center of LSD1. The fluorines of 2-PFPA stabilizes its phenyl ring position by restricting its torsion angle, thus preventing the phenyl ring from twisting past Y761 of LSD1 (Figure 3). When compared to the crystal structure of another LSD1 inhibitor *trans*-2-phenylcyclopropylamine hydrochloride (2-PCPA) (PDB: 2UXX), the residues around FAD, such as I356, V333, Y761, T335, L706, and F538 (Figure 3), had moved slightly away from the reactive cavity, suggesting that the reactive cavity creates more space to accommodate the extra fluorine atoms of PFPA [96]. The binding of 2-PFPA to LSD1 prevents the further binding of the substrate to FAD, thus inhibiting LSD1 demethylase activity. Based on reported X-ray crystal structures of LSD1, many LSD1 inhibitors have been identified using virtual screening. Zhang’s group screened nine potential LSD1 inhibitors with IC_50_ values in the micromolar level. Among them, compound XZ09 showed moderate selectivity for LSD1 over MAOs, but the in cellulo and in vivo activity of this compound was not further studied in their paper [25]. Sharma’s group used virtual screening to identify a compound, HCL-2509, with high selectivity and nanomolar potency (13 nM) [97]. This compound exhibited good in vitro and in vivo anti-cancer activity in mouse models of AML and Ewing sarcoma [97,98]. The compound also inhibited the proliferation of many cancer cell lines such as prostate cancer and neuroblastoma [97,99]. The low-cost and high-throughput method greatly improves screening efficiency by rapidly weeding out non-binders in silico. However, because virtual screening is based on the physicochemical properties of compounds rather than their biological activity, this technique may produce a high rate of false positives or false negatives [100,101]. Therefore, it is imperative to combine this method with other biochemical assays to verify the in silico results [25].

### 4.2. Substrate-Based Assay

Mass spectrometry (MS) can determine the presence and quantity of a specific molecule by obtaining its accurate mass, and it has been applied for screening LSD1 inhibitors [89,90]. Utilizing a short peptide corresponding to the first 21 residues of histone 3 as a substrate, the amount of unmethylated and mono-methylated peptides can be measured by MS after incubation with LSD1, which indicates the progress of LSD1 demethylation. Although this method is direct, exact, label-free, and widely applicable, its inapplicability for high-throughput screening and high requirements in terms of instruments and operators makes it only useful as an assisted screening method [89,90]. This technique is often combined with other high-throughput methods, such as virtual screening, to improve screening efficiency [102,103].

### 4.3. Byproduct-Based Assay

Enzyme-coupled assays are indirect assays for LSD1, as they quantitate the byproducts (H_2_O_2_ or HCHO) produced during the demethylation process by LSD1. Enzyme-coupled assays include the luminol coupled assay [91], Amplex Red-coupled assay [92], 4-aminoantipyrine-coupled assay [93], and formaldehyde dehydrogenase (FDH)-coupled assay [94]. These assays all rely on another enzyme to evaluate LSD1 demethylase activity. For example, the FDH-coupled assay quantifies production of NAD+ into NADH by detecting absorbance at λ = 340 nm, or via measuring fluorescence intensity at 460 nm with excitation at 330 nm [104]. On the other hand, the other three methods evaluate demethylase activity through detecting H_2_O_2_ production with the aid of the horseradish peroxidase (HRP) enzyme [91,105,106,107]. Although these four methods are label-free, low in cost, and suitable for high-throughput screening, the application of these techniques is restricted to the detection of compounds that do not interact with H_2_O_2_ [62,63,64,65] or HCHO [94]. Compounds autofluorescence or fluorescence quenching ability are also not suitable for these methods. Therefore, the chemical characteristics of compounds should be considered before screening.

### 4.4. PPI-Based Assay

Homogeneous time-resolved fluorescence (HTRF) and amplified luminescent proximity homogeneous assay are widely applied to study protein–protein, protein–peptide, and protein–DNA/RNA interactions in different stages of drug studies [108,109]. Both of these methods are based on fluorescence resonance energy transfer (FRET) and require specific antibody-coated beads. Recently, commercial kits using HTRF or ALPHA-based methods for LSD1 inhibitor screening have been launched, and have the advantages of low background, high sensitivity, and high reproducibility. However, there are several limitations of these techniques. First, the labeling of the proteins may induce undesirable conformational changes in the protein that may lead to false-positive or false-negative results in the screening. In addition, the technologies are susceptible to interference by compounds with intrinsic autofluorescence and/or fluorescence quenching ability [108,109]. Meanwhile, the scintillation proximity assay (SPA) is a radioisotope-based method that is widely used for high-throughput screening protein-peptide/DNA interaction assays [110]. In this assay, biotin-labeled methylated peptides are demethylated by LSD1, and the protein methyltransferase KMT7 is subsequently added to methylate the peptide product using ^3^H-labeled *S*-(50-adenosyl)-l-methionine (SAM). Therefore, the level of ^3^H incorporation into the target peptide can be correlated with LSD1 activity [111]. This method is very quick, sensitive and suitable for high-throughput screening. However, limitations of the method include the requirement for specialized apparatus for measuring and protecting against radiation exposure during inhibitor screening. To save cost and reduce the rate of false positives or false negatives, the combination of this method with virtual screening may be a good option.

## 5. Pharmacological Inhibition of LSD1 for Cancer Therapy

Due to the potential of LSD1 as an anti-cancer target, several LSD1 inhibitors have been explored, with some of these having entered clinical trials or even clinical use. Here, we group the inhibitors into five subcategories: MAO inactivators and their derivatives, natural products, peptide inhibitors, polyamine-based inhibitors, and metal-based inhibitors.

### 5.1. MAO Inactivators and Their Derivatives

**Figure 4:**
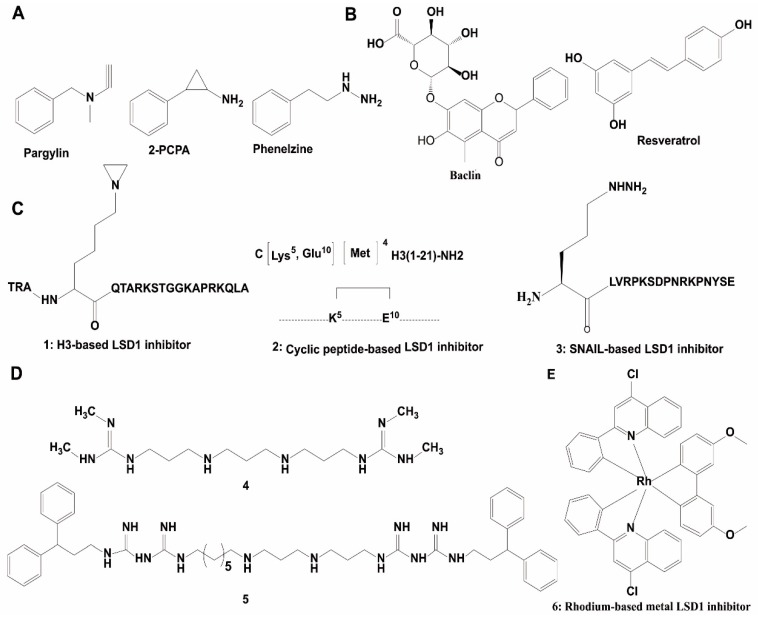
Chemical structures of representative LSD1 inhibitors. (**A**) Non-selective LSD1 inhibitor; (**B**) natural product-based LSD1 inhibitors; (**C**) peptide-based inhibitors; (**D**) polyamine-based LSD1 inhibitors; (**E**) metal-based LSD inhibitors.

Due to the significant similarity between LSD1 and MAO in their catalytic domains, early LSD1 inhibitors were developed based on the structural characteristics of the binding pocket of LSD1 and reported MAO inhibitors via virtual screening and/or chemical modifications [102,112]. Pargyline, tranylcypromine and phenelzine are three known MAO inactivators that have also shown inhibition of LSD1 demethylase activity (Figure 4A). *Trans*-2-phenylcyclopropylamine hydrochloride (2-PCPA), first identified as a MAO inhibitor, was also demonstrated to irreversibly inhibit LSD1 activity through forming a covalent bond to the FAD-binding motif. Therefore, 2-PCPA is a non-selective LSD1 inhibitor because it inhibits both MAOs and LSD1 [102,113,114,115]. To improve the selectivity of LSD1 inhibitors, numerous 2-PCPA derivatives have been synthesized, with some of these exhibiting good potency and high selectivity in aid of virtual screening. ORY-1001, an LSD1 inhibitor that has entered phase II clinical trials for AML, is an N-alkylated 2-PCPA derivative that possess an IC_50_ of 18 nM against LSD1 and selectivity over LSD2 and MAOs [116,117]. GSK2879552, another N-alkylated 2-PCPA derivative, is both selective for LSD1 and orally bioavailable. This compound inhibited the growth of small cell lung cancer (SCLC) cells in vitro and in vivo, and has entered clinical trials for SCLC treatments [118]. Some researchers have developed LSD1 inhibitors via mimicking the lysine group at the meta-position (NCL-1) or the para-position (NCL-2) of the phenyl ring, and the resulting compounds have exhibited improved inhibitory activities for LSD1 and selectivity for LSD1 over MAOs [114]. Mechanistically, NCL-1 and NCL-2 irreversibly inactivated LSD1 through forming a covalent bond with FAD, which induced the accumulation of H3K4me2, leading to the transcriptional upregulation of tumor suppressor genes and eventually repression of cancer cell growth [26]. Subsequently, NCL-1 derivatives were developed with the aim of improving their biological activity [112], and these analogues exhibited good anti-cancer activities in solid tumors, such as breast cancer [119], prostate cancer [120], and glioma [121,122] in cellulo and/or in vivo. Phenelzine is another scaffold that inhibits both LSD1 and MAOs. To improve the selectivity of this compound, a hybrid molecule with the scaffold of phenelzine and bizine was designed, which inhibited LSD1 with a K_i_ of 59 nM and also possessed higher selectivity for LSD1 over other enzymes containing the AOD domain [123]. This compound exhibited good anti-proliferation effect in cancer cell lines LNCaP and H460 [123].

### 5.2. Natural Products and Their Derivatives

Natural products offer a diverse array of chemical scaffolds with distinct activity profiles and relatively mild toxicity. Several natural products have been found with LSD1 inhibitory activity in vitro [124,125], such as baicalin, resveratrol and geranylgeranoic acid (GGA) (Figure 4B). Baicalin was the first reported flavonoid-based non-covalent LSD1 inhibitor (IC_50_ = 3.01 μM). The sugar moiety in baicalin was found to be critical for LSD1 inhibitory activity. Resveratrol, an irreversible LSD1 inhibitor in vitro and *in cellulo*, was postulated to inhibit LSD1 activity by directly binding to the protein [124,125]. GGA and its derivatives are exhibited inhibition of LSD1 activity. They impaired the LSD1-H3K4me2 PPI, leading to the transcriptional upregulation of the NTRK2 gene in SH-SY5Y cells [126].

### 5.3. Peptide-Based Inhibitors

The molecular size of peptides makes them ideal scaffolds for developing PPI inhibitors [127]. Many potent and selective peptide inhibitors were developed through consideration of the substrate-binding domain of LSD1. A 21-mer linear peptide (compound **1**), corresponding to the substrate region of LSD1 except with the fourth Lys being replaced by methionine, was a potent inhibitor of LSD1 (K_i_ = 0.04 μM) and against the LSD1-CoREST PPI (K_i_ = 0.05 μM) (Figure 4C) and [128]. Moreover, the cyclic peptide **2** was identified as a potent LSD1 inhibitor with an IC_50_ value of 2.1 μM and a K_i_ value of 385 nM against the LSD1/CoREST PPI (Figure 4C). It also exhibited moderate anti-cancer activity in MCF-7 and Calu-6 cell lines [129]. The LSD1-SNAIL interaction promotes cancer cell invasion and is a new target for cancer therapy [56]. A SNAIL peptide-based molecule (peptide **3**) (Figure 4C) has been reported as a selective LSD1 inhibitor with an IC_50_ value of 0.28 μM and anti-proliferation effect for Hele cells [130].

### 5.4. Polyamine-Based Inhibitors

The highly conserved structure and catalytic similarities of FAD-dependent oxidases, the strong affinity of polyamines with chromatin, and the structural similarity between polyamines and the lysine tails of histones, promoted researchers to explore whether polyamine analogues could be developed as LSD1 inhibitors [91]. Polyamine analogues were first identified as LSD1 inhibitors in 2007 (Figure 4D) [91]. Among them, compounds **4** and **5** showed significant inhibition against LSD1 in vitro and *in cellulo*. In HCT116 cells, these compounds inhibited LSD demethylase activity, thus leading to the accumulation transcription-activating H3K4me2/1, and thus leading to anti-cancer effects in vitro and in vivo [91]. In subsequent studies, derivatives of these compounds were found to inhibit cancer progression via enhancing expression of SFRP5 and GATA4, while downregulating Wnt signaling [106,131,132].

### 5.5. Metal Complex Inhibitors

The novel rhodium(III) complex **6** is the first metal-based inhibitor of LSD1 activity reported in the literature. This metal complex occupied the binding pocket of LSD1 for histone H3 recognition and thus blocked the LSD1-H3K4me2 interaction in human prostate cancer cells, leading to increasing amplification of p21, FOXA2, and BMP2 gene promoters. With an IC_50_ of 0.04 ± 0.008 μM for LSD1, this complex was selective for LSD1 over MAOs and also showed anti-proliferative activity toward human cancer cells (Figure 4E) [133]. However, further work needs to be done to improve the bioavailability of the rhodium(III) complex in vivo.

### 5.6. Others

With the development of computer-aided drug screening, several selective and novel LSD1 inhibitors were reported. CBB-1007 is a reversible, potent, and cell-penetrating LSD1 inactivator (IC_50_ = 5.3 μM) [134]. GSK690, also known as GSK354, inhibits LSD1 (IC_50_ = 90 nM) with selectivity over other enzymes with AOD domains, and has shown effectiveness against AML with low cytotoxicity to animal cells [135,136,137]. HCl2509 (also named SP2509), a non-covalent LSD1 inhibitor with a benzohydrazide scaffold, inhibits LSD1 with an IC_50_ of 13 nM and has specificity for LSD1 over MAOs [35]. This compound showed the ability to efficiently reduce cell growth in prostate cancer, endometrial cancer, and Ewing sarcoma [71,99,138,139].

## 6. Concluding Remarks and Perspectives

Although extensive research on LSD1 has been performed, and parts of its roles in cancer progression and development have been elucidated, the precise functions of LSD1 in cancer stemness, drug resistance and autophagy are yet to be investigated. As an oncogene, LSD1 attracts tremendous attention from medicinal chemists. To develop new LSD1 inhibitors, efficient screening methods are required for their detection. However, the use of only a single screening method may be insufficient for identifying new LSD1 inhibitors, due to the high rate of false-positive or -negative results caused by inherent drawbacks of the screening methods (Table 2). Therefore, a successful screening campaign for LSD1 generally needs to combine at least two types of orthogonal assays. For example, a byproduct quantification-based assay can be used as a primary screen, followed by validation of the hits using a substrate quantification-based assay such as a FRET-based assay or mass spectrometry. In addition, surface plasmon resonance (SPR), isothermal titration calorimetry (ITC) and bio-layer interferometry (BLI) can also be used to investigate the parameters of the drug-LSD1. The ready availability of LSD1 co-crystal structures also enables the use of virtual screening techniques to rapidly prioritize likely binders for synthesis and screening. Finally, cell and/or animal-based assays should be performed to identify LSD1 inhibitors with bioactivity *in cellulo* and in vivo.

Although several types of LSD1 inhibitors have been documented in the literature, with some even undergoing clinical trials, there are still many problems that have to be overcome before LSD1 inhibitors can reach the clinic. Firstly, although virtual screening against the LSD1 catalytic pocket has been used to develop many LSD1 inhibitors, the high similarity of LSD1 with LSD2 and MAOs makes it more difficult to develop selective inhibitors of LSD1 [124,125]. One way to overcome this is to target the other domains that are involved in allosteric regulation of LSD1 demethylase activity, rather than the converted catalytic domain. Since most reported LSD1 inhibitors focus on targeting the catalytic machinery, the investigation of molecules targeting allosteric domains may yield new scaffolds with exquisite selectivity. In addition, metal-based complexes may be another choice for achieving potent and selective LSD1 inhibitors. Metal complexes are characterized by their stability, distinct geometries, and diverse structures, making them suitable as chemical scaffolds for exploring the binding sites of proteins [133,140,141,142,143,144]. For example, our group has identified a rhodium(III)-based inhibitor with potency and selectivity for LSD1 in vitro and *in cellulo* [133]. Another approach to generating LSD inhibitors is to use substrate analogues to disrupt the natural LSD-peptide PPI based on the structural features of the substrate binding pocket of LSD1 [91,127]. Secondly, it is important to note that although many LSD1 inhibitors could abrogate LSD1-mediated demethylation, they may not necessarily show potent anti-cancer activities, as many oncogenes or tumor suppressor genes are often regulated by multiple enzymes [100]. To tackle this problem, the combination of LSD1 inhibitors with other drugs may be a viable solution. HDAC1 has been reported to form complex with LSD1 and inhibit the transcription of tumor-suppressed genes. Therefore, the dual administration of LSD1 and HDACs effectively inhibited the proliferation of many cancer cell lines [145]. LSD1 inhibition also enhanced PD-(L)1 blockade-induced anti-tumor immunity [146]. The combination therapy of an inhibitor of enhancer of zeste homolog 2 (EZH2), a histone-lysine *N*-methyltransferase enzyme that is upregulated in multiple cancers and promotes tumorigenesis, and LSD1 exhibited unexpected synergistic effects against AML in vitro and in vivo [147]. These researches indicate that combination therapy could be a feasible strategy for overcoming the low individual potency of LSD1 inhibitors. Additionally, the potential side effects of LSD1 inhibition on the blood is also an important concern [148]. Previous research has shown that LSD1 knockout perturbs the formation of blood cells (granulocytes and red blood cells), which would lead to acute anemia and a decrease in the numbers of platelets [75]. Some LSD1 inhibitors have also been reported to impair erythropoiesis [75,148,149]. Interestingly, the LSD1 inhibitor tranylcypromine did not exhibit toxicity when used for the treatment of neurological disorders in mice or human beings [150]. Therefore, the adverse effects on erythropoiesis should be addressed in preclinical and clinical evaluation of a new LSD1 inhibitor. A final challenge for bringing LSD1 inhibitors into the clinic is the fact that LSD1 plays a different role in different cancers. Therefore, scientists need to work closely with oncologists to identify which cancers are sensitive to treatment with LSD1 inhibitors in vivo. While this review has summarized the recent works that have contributed to our understanding of LSD1 biology and inhibition, there is still a long way to go before the cancer adaptation profile of LSD1 inhibitors is determined and the inhibitors are widely applied in these human cancers.

## Figures and Tables

**Table 1:** LSD1 substrates and regulatory functions.

Type of Substrates	Protein	Subunits of Complex	Functions
Histone	H3K4me2/me1	RCOR2	Regulates ESC property and reprogramming somatic cells to pluripotency
		NuRD (MTA3)	Nucleosome remodeling and H3K4 demethylation
		TLX	Regulation of neuronal stem cell proliferation
		CtBP/CoREST	Repression of growth hormone expression in hypophysis
		HDAC/CoREST	Regulation of pluripotency of embryonic stem/carcinoma cells
		HDAC/SIN3A	Maintenance of sensitivity to chemotherapy in breast cancer
		Core-BRAF35	Mediates repression of neuronal specific genes in nonneuronal tissues
		HP1/SU(VAR)3–9	Heterochromatic gene silencing
		HOTAIR/PRC2	HOX gene silencing
	H3K9me2/me1	ERα	Activation of estrogen receptor alpha-dependent genes
		spLSD1/2	Control of replication, imprinting and heterochromatin propagation
		AR	LSD1 functions as an activator of androgen receptor-responsive genes
Non-histone	p53	-	Suppresses tumor and activates transcription
	DNMT1	-	Maintains DNA methylation
	E2F1	-	Regulates DNA damage-induced stabilization and apoptotic function.
	HIF-1α	-	Regulates transcription of VEGF
	STAT3	-	Regulates gene expression through the formation of dimers

**Table 2:** Comparison of different screening methods for LSD1.

Category	DetectedSpecies	Methods	Benefits	Drawbacks
Target-based assay	LSD1	Virtual screening	Low cost; High-throughput	High false positive rate; only applicable for primary screening
		SPR	Label-free; low false positive rate	Low-throughput; high technical and equipment requirements; high cost
		ITC		
		BLI		
Substrate-based assay	Truncated H3	MS-based assay	Label-free; direct detection of product	Requirement of expensive instrumentation, dedicated operation professionals, and complicated sample preparation steps
Byproduct-based assay	H_2_O_2_	Luminol couple assay	Low cost; high-throughput; label-free; high sensitivity	Only applicable for compounds that do not interact with H_2_O_2_
		Amplex red- coupled assay		Only applicable for compounds that do not interact with H_2_O_2_ and without autofluorescence or quenching ability
		4-aminoantipyrine-coupled assay	Low cost; high-throughput; label-free	Low sensitivity; Only applicable for compounds that do not interact with H_2_O_2_
	HCHO	FDH-coupled assay		Only be applicable for compounds that do not interact with HCHO
PPI-based assay	LSD-peptide PPI	HTRF-based assay	High-throughput, easy to operate; high sensitivity	Labeling requirement; interference by compounds with autofluorescence or fluorescence quenching ability
		HTRF-based assay		
		Scintillation proximity assay	high-throughput; high sensitivity	Heat required; special instrument required; additional enzyme introduced
